# Postharvest Physiology and Handling of Guava Fruit

**DOI:** 10.3390/foods13050805

**Published:** 2024-03-06

**Authors:** Nanhui Chen, Wei Wei, Yingying Yang, Lin Chen, Wei Shan, Jianye Chen, Wangjin Lu, Jianfei Kuang, Chaojie Wu

**Affiliations:** State Key Laboratory for Conservation and Utilization of Subtropical Agro-Bioresources/Guangdong Provincial Key Laboratory of Postharvest Science of Fruits and Vegetables/Engineering Research Center of Southern Horticultural Products Preservation, Ministry of Education, College of Horticulture, South China Agricultural University, Guangzhou 510642, China; 13318879635@163.com (N.C.); weiwei_11663@163.com (W.W.); yangyy1992@scau.edu.cn (Y.Y.); chenlin_304@scau.edu.cn (L.C.); shanwei@scau.edu.cn (W.S.); chenjianye@scau.edu.cn (J.C.); wjlu@scau.edu.cn (W.L.); jfkuang@scau.edu.cn (J.K.)

**Keywords:** shelf-life extension, physical preservation, CA storage, packaging, chemical treatment

## Abstract

Guavas are typical tropical fruit with high nutritional and commercial value. Because of their thin skin and high metabolic rate, guavas are highly susceptible to water loss, physical damage, and spoilage, severely limiting their shelf-life. Guavas can typically only be stored for approximately one week at room temperature, making transportation, storage, and handling difficult, resulting in low profit margins in the industry. This review focuses on the physiological and biochemical changes and their molecular mechanisms which occur in postharvest guavas, and summarizes the various management strategies for extending the shelf-life of these sensitive fruits by means of physical and chemical preservation and their combinations. This review also suggests future directions and reference ideas for the development of safe and efficient shelf-life extension techniques.

## 1. Introduction

Guava (*Psidium guajava* L.; Myrtaceae) is an economically important fruit produced in tropical and subtropical regions, including South China, India, Pakistan, Mexico, and Brazil. In most African countries, guava is regarded as a minor crop, except for in South Africa, Egypt, and Sudan, which have invested in the further study of guava in areas such as genetic breeding and disease management [1]. In recent years, Kenya has produced more than 11,000 tonnes of guava annually, worth USD 1.1 million, and production is increasing according to the Kenya Horticultural Crops Agency [2]. Guavas possess considerable nutritional and medicinal value, being rich in soluble sugars, proteins, dietary fibers, and vitamins. In addition, guavas contain diverse bioactive compounds, including tannins, flavonoids, pentacyclic triterpenoids, carotenoids, and polyphenols. These bioactive constituents have been found to provide anticancer and anti-inflammatory benefits, protect the heart and eyes, and regulate blood sugar [3,4,5].

Guavas are plagued by a number of postharvest quality issues owing to their thin skins, including pathogen invasion, water loss, and rapid textural decline. Moreover, guavas are climacteric fruit which often reach peak respiration shortly following harvest, resulting in a short storage life: approximately one week at room temperature or two weeks at 6–8 °C in strict atmosphere conditions [6,7]. In addition, as tropical fruit, guavas are sensitive to chilling injury during transport or storage. A short shelf-life, mechanical damage, and postharvest loss are considered the major factors limiting the modern guava industry [7].

Guavas are alive or metabolically active after harvest and during storage, consuming nutrients through respiration and transpiration. Respiration, ethylene production, transpiration, chilling injury, and infection with spoilage organisms all negatively impact shelf-life, storage quality (i.e., weight loss), pathogen susceptibility, and texture (e.g., browning, softening, and decay). In addition, guavas lose sugars, phenols, free amino acids, soluble proteins, flavonoids, and ascorbic acids during storage [8].

### 1.1. Respiration

Respiration involves the decomposition of complex organic matter (e.g., carbohydrates, proteins, and fats) into smaller molecular substances and ultimately into CO_2_ and H_2_O, accompanied by energy production and heat release. Due to biochemical and structural factors, most guava varieties exhibit a climacteric respiratory peak during the ripening or aging period. Biochemically, CO_2_ produced through the decarboxylation of malic acids couples with phosphorylation and rapidly increases the respiratory rate. Structurally, the disintegration and aging of chloroplasts following harvest increases enzymatic activity and accelerates ethylene synthesis, again increasing the respiratory rate and shortening shelf-life [9]. Climacteric guavas are typified in the ‘Li-Tzy Bar’ (‘LTB’) variety, while non-climacteric guavas, which exhibit ethylene auto-catalysis and short shelf-life, are typified in the ‘Jen-Ju Bar’ (‘JJB’) variety. The non-climacteric cultivar showed good stability at low rates of respiration (19.8–7 mg CO_2_ kg^−1^ h^−1^), while the climacteric one indicated high respiratory rates (171 mg CO_2_ kg^−1^ h^−1^ at climacteric peak) [10]. The respiratory rate of guava is significantly lower than other varieties such as papaya and banana (20–30 mg CO_2_ kg^−1^ h^−1^), but significantly higher than that of many varieties (60–80 mg CO_2_ kg^−1^ h^−1^ and 120–150 mg CO_2_ kg^−1^ h^−1^, respectively) when the respiratory peak comes.

### 1.2. Ethylene Biosynthesis

The phytohormone ethylene regulates fruit ripening and senescence by accelerating respiration, ripening, and chlorophyll decomposition. Ethylene is synthesized through the methionine (Met) cycle, in which ACC synthetase (ACS) catalyzes the formation of 1-aminocyclopropyl-1-carboxylic acid (ACC) from S-adenosylmethionine (SAM) and ACC oxidase [ACO, also known as ethylene synthetase (EFE)] catalyzes the formation of ethylene from ACC [11]. The ethylene biosynthesis system can be divided into system-1 and system-2. System-1 is responsible for the production of low and basal concentrations of ethylene in climacteric and non-climacteric fruits, and is regulated by negative feedback and/or self-inhibition. System-2 is responsible for the production of large amounts of ethylene during the ripening of climacteric fruits, and is regulated by positive feedback and/or self-catalysis [12].

Recent research suggests that system-2 defects in non-climacteric guava cultivars are mainly the result of the silencing of the ethylene biosynthesis gene *PgACS1*, which governs the ripening period, and *PgACS2*, which controls fruit set. In addition, the functioning of system-2 is also affected by the downregulation of *PgACO1* and *PgACO2* [10].

Because ethylene is a gaseous molecule, ripening and senescence can be regulated through atmospheric control during storage. Specifically, ethylene production can be inhibited by the application of CO_2_, silver thiosulfate (STS), and 1-methylcyclopropene (1-MCP).

### 1.3. Transpiration

Because guavas continue to transpire following harvest, they lose both nutrients and water during storage. In addition, as the intracellular fluid becomes more concentrated, the resulting relative increase in ionic content can result in cellular poisoning. Postharvest water loss can cause loss of turgor pressure, stomata closure, and metabolic disturbance, all of which result in tissue degradation, wilting, shrinking, and softening. These textural changes indicate declining fruit quality and can lead to commercial devaluation.

In guavas, transpiration occurs through channels in the cuticle. Because guavas have thin cuticles with little wax, postharvest transpiration can be reduced by controlling storage conditions. This includes maintaining high relative humidity (RH) and air pressure, as well as low temperature, air flow rate, and light [13].

### 1.4. Chilling Injury

Guavas are prone to chilling injury when stored at temperatures below 6 °C for a certain duration [14]. The symptoms of chilling injury in guava fruit include wrinkling, pitting, lignification, browning and/or softening of the fruit surface or flesh, flooded patches, failure to mature, increased sensitivity to fungal decay, and rapid decomposition. Above all, browning is considered the most typical symptom of CI. Cold damage can lead to the destruction of the internal structure of fruits and vegetables, the loss of nutrients, and a decline in disease resistance and storage resistance, and the subsequent deterioration of fruits and vegetables. Studies have indicated that low-temperature stress triggers the production of excessive intracellular reactive oxygen species (ROS), resulting in oxidation of macromolecules such as DNA, proteins, and lipids, as well as damage to the plasma membrane, weakening its selective permeability [15]. These disturbances increase cellular disorder and decrease fruit quality.

At room temperature, guavas typically exhibit a 3–4 d shelf-life, although at suitably low temperatures (7–10 °C), the shelf-life can be extended to 2–3 weeks [16,17,18]. However, the threat of chilling injury has restricted the use of cold transport and storage, which has greatly impacted the economic value of guava fruit.

### 1.5. Postharvest Spoilage

Mechanical wounding of guava fruit before, during, and after harvest results in rapid invasion by spoilage microorganisms and decay. Damaged fruit exhibit black or brown pitting, peel browning, and tissue softening. Some of the most important fungal infections affecting guava fruit quality are anthracnose (*Colletotrichum* spp.) and soft rot (*Aspergillus flavus* and/or *Rhizopus stolonifer*) [19,20]. The most common fungal spoilage organisms include species of the *Fusarium* and *Aspergillus* genera [21]. In addition, guavas can become susceptible to decay caused by bacteria and other microorganisms, including *Bacillus megaterium*, *B. subtilis*, *B. cereus*, *Enterobacter aerogenes*, *Micrococcus luteus*, *Klebsiella pneumoniae*, *Staphylococcus aureus*, *S. epidermidis*, and *Proteus vulgaris* [22].

## 2. Physical Postharvest Preservation of Guava Fruit

Recent studies point to several promising techniques for extending the shelf-life of guava fruit, including controlled atmosphere storage, modified packaging, and physical and chemical treatments.

### 2.1. Controlled Atmosphere Storage

Controlled atmosphere (CA) storage, also referred as to air conditioning storage, implies regulating the composition, concentration, and pressure of O_2_, CO_2_, ethylene, and other gases in the storage environment, up to and including imposing low-O_2_ (LO) conditions [23,24]. A subtype of CA is modified atmosphere (MA) storage. Specifically, CA utilizes mechanical gas regulation and monitoring to artificially control the composition and concentration of gases in a storage environment. The relatively closed nature of the storage environment ensures that the gases are accurately monitored and regulated to produce optimal storage conditions. However, CA warehouses are costly to construct and operate, making them unpopular among food business operators. MA, also known as simple gas regulation or limited gas storage, relies on the physiological capacity of the fruit (i.e., respiration and ethylene production) to spontaneously regulate the concentration of O_2_ and CO_2_ under certain ventilation capacities. MA has gained popularity among food business operators due to its relatively low investment cost and ease of operation.

Long-term CA storage (including LO and/or high-CO_2_ conditions) can adversely affect fruit quality by inducing the production of ethanol and acetaldehyde, undesirable odors, and ROS, and promoting over-maturation and nutrient loss [25]. For example, Teixeira et al. [26] reported that guava (cv. ‘Pedro Sato’) fruit quality was negatively affected after 28 d of storage at 12.2 °C under 5% O_2_ and elevated CO_2_ (10%, 15%, and 20%), even though there was no difference in the respiratory rate between fruit stored under ambient and modified atmospheric conditions. Perhaps most notably, fruit firmness dropped sharply under 5% O_2_ and 20% CO_2_ conditions, and the soluble pectin content increased on the 14th d of storage [26]. Kader [27] reported that the optimal storage conditions for guava are between 2–5% O_2_ and 0–1% CO_2_ at 5–15 °C. Storage at 40% CO_2_ and <1% O_2_ for 12 h has been shown to curtail insect growth, maintain quality, and lengthen the shelf-life of guava fruit [28]. Brackmann et al. [29] found that guavas (cv. ‘Paluma’) decayed more slowly when stored under different CA conditions compared with those stored under ambient atmospheric conditions. Finally, pre-treatment with nitric oxide (N_2_O) successfully inhibits the development of decay in guava [30].

### 2.2. Refrigerated Storage

Refrigeration is the most common and effective storage practice for extending the shelf-lives of many fruits and vegetables, including citrus [31], pineapple [32], apple, and pear [33]. A gradual cooling method is preferred for particularly cold-sensitive fruits. For example, Singh and Pal [34] report that the shelf-life of guava could be extended to 30 d under 8 °C storage. However, because the temperature of commercial cold storage warehouses tends to fluctuate by about 5 °C, refrigerated guavas are still susceptible to chilling injury. In guavas, the minimal optimal storage temperature depends on both variety and maturity status.

### 2.3. Packaging

#### 2.3.1. Controlled and Modified Atmosphere Package

Both CA packaging (CAP) and MA packaging (MAP) rely on the creation of an artificial and relatively closed storage environment around the fruit in order to extend its shelf-life. Generally, the packaging consists of a thin plastic film, which can be manufactured from high-density polyethylene (HDPE), polypropylene (PP), polyvinyl chloride (PVC), polyvinyl alcohol (PVA), or low-density polyethylene (LDPE) [35]. However, plastic packaging, especially LDPE and PP, results in the production of environmentally sustainable, difficult-to-degrade plastic waste [9,26,36]. MAP is dynamic and self-regulated, and relies on the permeability of the film to adjust the gas composition and control ethylene production during storage [9]. MAP delays the loss of bioactive compounds, including phenols and flavonoids, and maintains the antioxidant capacity of the fruit [37]. On the other hand, CAP precisely regulates the atmosphere environment through the use of sensors or electronic controls.

Both MAP and CAP can significantly improve the shelf-life and maintain the sensory qualities and nutritional profile of guava fruit (Table 1). However, to date, the majority of studies have focused on the use of MAP to maintain postharvest quality in guava. For example, wrapping guava fruit in tissue paper has been shown to extend the shelf-life to 12 d at 16 °C compared to 24 °C, effectively preventing water loss and maintaining physiological and biochemical characteristics [38]. Similarly, the use of MAP has been shown to extend the shelf-life of guava fruit to 7 d at 25 °C [39]. Guavas packaged in LDPE with 9% O_2_ and 5% CO_2_ can be preserved for 21 d at 10 °C and 7 °C [36,40,41]. Similarly, guavas packaged in PP can be preserved up to 28 d at 8–12 °C [17]. Finally, Kumar et al. [42] report that PP-based MAP could extend the shelf-life of guava fruit to 25 d at 6 °C. The optimal CAP storage conditions for guava fruit remain to be clarified.

#### 2.3.2. Edible Packaging

Edible packaging has recently been introduced in order to protect the environment and enhance food safety. Edible packaging, generally in the form of films and coatings, can be made from combinations of polysaccharides, proteins, and lipids [43]. Polysaccharide-based edible polymers are commonly made of corn starch, tapioca starch, potato starch, cellulose, hemicellulose, and/or gums; protein-based edible polymers are primarily based on either casein or zein; and lipid-based edible polymers include oils and waxes. In films, polymers comprised of independent material are used to cover the food surface, while coatings are formed directly on the food surface by impregnation or spraying [44]. Edible packaging relies on the water-repellent and air-blocking properties of the polymers to create a barrier between the food and the external environment. As with other forms of packaging, these barriers decrease the respiration, transpiration, surface scarring, and invasion of pathogenic or spoilage organisms.

Researchers have tested the performance of many edible polymers on postharvest guava quality and shelf-life (Table 2). Hong et al. [45] found that a 2.0% (*w*/*v*) chitosan solution in combination with low temperature (11 °C) could significantly delay ripening and maintain quality, likely because chitosan can reduce oxidative stress and protect cell membranes from damage. Francisco et al. [46] reported that a biodegradable film made of 25% acetylated cassava starch (ACS) and 75% hydroxyethyl cellulose (HEC) could maintain the coloration and firmness of guava fruit for 13 d. The researchers explained that HEC75 was able to maintain these quality parameters due to a combination of solubility, opacity, water vapor transportation, thickness, and other beneficial characteristics [46]. Vishwasrao and Ananthanarayan [47] found that a coating containing 1% hydroxypropyl methycellulose (HPMC) and 0.3% palm oil (PO) substantially reduced weight loss, softening, and color change while maintaining the antioxidant capacity and nutrient profile of guava fruit stored for 9 to 12 d at 24 ± 1 °C and 65 ± 5% RH. Both tannic acid-cross-linked zein olatin coating (ZTA) and non-cross-linked zein olatin coating (Z) have been shown to improve the stability of guava fruit during storage [48]. Both Oliveira et al. [49] and Formiga et al. [50] showed that biopolymeric coatings hydrophobized with beeswax (BW) and HPMC composite coatings could delay fruit ripening, maintain fruit firmness and color, and extend the shelf-life due to their low rates of water vapor transmission and O_2_-CO_2_ exchange.

In addition, plant extracts used in edible packaging can extend the shelf-life of guava by affecting the internal regulation mechanism of the fruit. Vichaiya et al. [51] showed that an exogenous trehalose coating could reduce cold damage in guava fruit by upregulating the expression of *SnRK1*. Nair et al. [52] reported that the introduction of pomegranate peel extract (PPE) into a chitosan- and alginate-based coating not only improved the sensory characteristics of guava, but also retained the nutritional value by reducing respiratory rate and aging. Rehman et al. [53] found that an aloe vera (AV) gel coating can delay ripening and reduce lipid peroxidation by maintaining antioxidant enzyme (APX, CAT, and SOD) activity and redox homeostasis. Finally, De Oliveira et al. [54] explored the effects of a composite coating of chitosan (Chi) and citronella citrate essential oil (CCEO) on postharvest quality of guava, and found that the Chi-CCEO coating effectively maintained postharvest quality over 10 d of storage at low temperature.

**Table 2:** Edible packaging on guava.

Cultivar	Coating Material	Main Findings	References
‘*Pearl*’	0.5, 1.0, and 2.0% chitosan.	2.0% chitosan coating significantly reduced firmness loss and water loss, and enhanced the antioxidant ability of fruit.	[45]
Unknown	100% acetylated cassava starch (ACS) + 0% hydorxyethyl cellulose (HEC), 75% ACS + 25% HEC, 50% ACS + 50% HEC, 25% ACS + 75% HEC, 0% ACS + 100% HEC.	75% HEC and 25% ACS or 100% HEC films delayed the firmness loss and color change, reduced ripeness for 13 d at 21.0 ± 1.2 °C with 53 ± 16% RH, and lengthened the shelf-life.	[46]
‘*Lalit*’	1% hydroxypropyl methyl cellulose (HPMC) and 0.03, 0.3% palm oil (PO).	1% HPMC and 0.3% PO delayed weight loss and color change, reduced the activity of oxidase, and lengthened the shelf-life to 12 d at 24 ± 1 °C and 65 ± 5% RH.	[47]
*Red guava*	Unmodified zein (Z) and zein treated with tannic acid (ZTA).	ZTA acted better when decreasing gas permeability, reducing respiration rates and ROS production, delaying the ripening process, and enhancing guava stability at 23 ± 2 °C and 88 ± 5% RH.	[48]
‘*Paluma*’	Composite coating based on Amylose and Amylopectin, 3% cornstarch, 2% cassava starch and 5% gelatin with beeswax (0, 5, 10%) and surfactant (0, 2.5, 5%).	The coating with 10% beeswax presented the best effects in the water vapor transmission rate (WVTR), enhancing the resistance ability and reducing weight loss at 15 ± 2 °C, 90 ± 2% RH.	[49]
‘*Pedro Sato*’	Hydroxypropyl methylcellulose (HPMC) +10, 20, 40% (dry basis) beeswax (BW).	HPMC + 20% BW acted the best, maintaining the quality and lengthening the shelf-life for 6 d at 21 ± 0.3 °C and 77 ± 6% RH.	[50]
‘*Kim Ju*’	0 and 200 mM of trehalose immersion for 30 min.	Exogenous trehalose induced a transient rise in sucrose against chilling injury.	[51]
‘*Allahabad Safeda*’	1% chitosan + pomegranate peel extract (PPE), 1% chitosan, 2% alginate + PPE, 2% alginate immersion for 1 min.	Chitosan with PPE showed the best effect on maintaining the overall quality and enhancing antioxidant ability of fruit, and lengthened the shelf-life to at least 20 d at 10 °C with 90 to 95% RH without chilling injury.	[52]
‘*Gola*’	0, 20, 40, 60, 80% aloe cera (AV) gel with AsA (4% *w*/*v*) as an antimicrobial agent, CaCl_2_ (3% *w*/*v*) as a firming agent, glycerol (1% *v*/*v*) as a plasticizer, and CMC (3% *w*/*v*) as a thickening agent added to each solution, dipped for 3 min.	AV gel coating, especially at high concentrations, presented better effects on the maintenance of commercial and medical value, along with a higher level of antioxidant capacity at 23 ± 2 °C and 70 to 75% RH.	[53]
Unknown	Bioconjugate sprays of GOx (glucose oxidase)/ZnONPs (zinc oxide nanoparticles), GOx/AgNPs (silver nanoparticles), ZnONPs, and AgNPs.	GOx/ZnONPs actively maintained biochemical quality and enhanced antioxidant system inside of fruit at 25 °C.	[55]

#### 2.3.3. Composite Packaging

The effects of other types of packaging, including antibacterial packaging, antifungal packaging, and nano packaging, have primarily been studied in the context of composite packaging comprised of some combinations of MAP, CAP, and/or edible packaging. For example, Shouket et al. [55] studied combinations of enzymes (glucose oxidase, GOx) and metal nanoparticles (silver, Ag; zinc, Zn) on the postharvest quality of guava fruit. They concluded that the GOx/ZnONP and GOx/AgNP combination sprays could effectively extend the shelf-life of guava fruit.

#### 2.3.4. Mechanical Packaging

Mechanical packaging is mainly used during transportation to reduce vibration. Mechanical packaging, such as foam boxes, and pearl cotton (EPE) mesh sets, disperses the impact force through the buffer to prevent the fruit from bruising that not only accelerates the respiratory rate, browning, and post-ripening, but also pave the way for microbial and pathogenic invasion. Chaiwong et al. [56] enhanced the biodegradability of natural rubber latex foam mesh (NRL-FN) buffer material by adding bamboo leaf fiber (BLF), which is an environmentally friendly cushion with excellent buffer capacity and designable properties.

### 2.4. Physical Treatment

Physical treatments include heat, ultraviolet radiation, ultrasonication, and combinations of these. Among these, heat treatments (HTs) are considered fairly conventional and have been in use for many years, while the other treatment options have emerged only recently. HTs can take many forms, including hot water dip (HWD), brief hot water rinsing and brushing (HWRB), hot air (HA), steam or vapor heating treatment (VHT), and radio frequency (RF) heating [57]. To make full use of the treatment and avoid damage, the temperature is generally controlled between 35 and 55 °C (Table 3). Guava fruits were treated by HW at 45 °C, 50 °C, and 55 °C for 3 min and it was confirmed that the measure of 45 °C maintained the postharvest quality best [58]. Research suggests that HTs can reduce respiration, ethylene biosynthesis, and the accumulation of toxic and cellular materials, effectively delaying ripening and extending the shelf-life of guava fruit. Microcosmically, HTs disrupt the functioning of several key enzymes, including ethylene-forming enzyme (EFE) (which coverts ACC to C_2_H_4_), pectin methylesterase (PME), and polygalacturonase (PG) (which initiates the pectin degradation and softening). HT also influences protein synthesis, secondary metabolism, and antioxidant enzyme activity [59,60,61,62]. Finally, HT is bactericidal and insecticidal [63]. Studies have indicated that VHT at 46 °C for 10 min can kill fruitfly larvae [64], HW at 47 °C for 20 min efficiently controlled anthracnose disease [65], and HW at 50 °C for 10 and 30 min can reduce microorganisms on fresh-cut guava [66]. Although HTs have been utilized to improve the shelf-life of guava fruit, conditions which may be suitable for some other fruits may be detrimental to guava due to its comparably thin skin. For example, 60 min of HT at 48 °C is acceptable for mango, but unsuitable for guava [67].

Ultraviolet (UV) radiation refers to the bands in the electromagnetic spectrum between X-rays (200 nm) and visible light (400 nm). UV radiation is characterized as long wavelength (UV-A, 320–400 nm), medium wavelength (UV-B, 280–320 nm), and short wavelength (UV-C, 200–280 nm) [68]. Among these, UV-C wavelengths are mainly used in postharvest management [69]. The main functions of UV-C treatment are surface sterilization [70,71], ethylene degradation, and induction of plant resistance [71,72,73,74]. Research suggests that the effects of UV-C may arise due to a combination of mechanical effects resulting from transient temporal collapse, such as high heating and cooling rates (109 K/s), shock wave formation, high temperature (5000 K), and high pressure (1000 atm). In microbes, these effects rupture cellular envelopes and photosensitize DNA even after very short exposures [75]. The antimicrobial effects of UV-C exposure are linked to the formation of cyclobutane thymine dimer and OH^−^/H^+^ chemistry, both of which can inhibit the growth of (or kill) microbes [76]. For example, exposure to UV-C radiation at an intensity of 16 KJ·m^−2^ can kill 100% of the eggs of *Ceratitis capitata* [77].

Ultrasonication (US) produces a pressure wave beyond human hearing (20–100 kHz) which induces acoustic cavitation. Cavitation results in the production of ROS in fruits and vegetables, leading to the inactivation of microbial contaminants and the stimulation of secondary metabolism [78,79,80].

The combination of UV-C and US has been shown to improve the antimicrobial capabilities of both [81], likely due to the low UV-C penetration of opaque and the high real-time energy consumption of production. Alterations to the US field promote cavitation and uniform UV-C exposure at the fruit surface. In addition, the use of agitation and mixing in a free-flowing US bath can reduce the standing wave effect and the generation of cavitation bubbles of different sizes to ensure uniform cavitation [80,82]. By changing the US frequency, variation in UV-C intensity can be reduced, allowing the radiation to reach the fruit surface more efficiently. Notably, synergies have been reported between US and chemical disinfectants, surfactants, organic acids, and/or electrolyzed water related to both surface decontamination and quality improvement [83,84,85]. For example, antimicrobial effects against *Cronobacter Sakazaki* have been reported in guava fruit subjected to US at 37 kHz and 380 W in combination with NaOCl application [86].

Overall, physical treatments are appealing to many producers and consumers because of their safety and effectiveness, but their application is still in the preliminary stage. In particular, the effects and mechanisms associated with combined treatments require further validation. The development and optimization of physical treatments will pave the way for improved postharvest management of guava and other sensitive commodities.

## 3. Chemical Treatments

### 3.1. 1-Methylcyclopropene (1-MCP) Treatment

1-MCP is a colorless, odorless, and non-toxic gas widely used in agriculture to delay ripening and aging in fruits and vegetables by competitively binding to ethylene receptors. 1-MCP indirectly and directly affects postharvest disease resistance by modulating ethylene signal transduction and induced systemic resistance (ISR), thus extending the shelf-life of fruits and vegetables [87]. 1-MCP treatment is often combined with CA storage, CA packaging, or edible packaging to delay the respiratory peak and ripening of fruits. Low concentrations (300 nL·L^−1^ 1-MCP) and short treatment durations (6 h) do not effectively delay the respiratory peak of guava fruit, as ethylene signaling is incompletely blocked. Nevertheless, higher concentrations (600 nL·L^−1^ 1-MCP) and longer treatment durations (12 h) have proven effective at extending the shelf-life of guava fruit to 25 d at 10 °C or 9 d at 25–29 °C [34].

### 3.2. Calcium Salt Treatment

When fruits are immersed in a calcium salt solution, the calcium ions enter the cell walls and form stable compounds, thereby improving the structural stability of the cell walls and slowing softening and aging. Exposure to calcium salts also improves the shelf-life by reducing respiration, protein catabolism, and spoilage susceptibility. However, through fruits and vegetables treated with calcium salts are considered safe for human consumption, calcium ions and other substances on the fruit surface may not be suitable for direct consumption. Calcium salt treatment has shown promise for maintaining the postharvest quality of guava fruit [88,89,90]. According to Deepthi et al. [91], 5–10 min of treatment with 2% calcium nitrate could extend the shelf-life of guava fruit (cv. ‘Lucknow-49’) to 23.83 d under cold storage (10 ± 10 °C and 90 ± 5% RH).

Calcium salt treatment can also be used in combination with other treatments to extend the shelf-life of guava fruit. For example, a combination of calcium chloride treatment and lemon grass fumigation significantly increased the content of ascorbic acid (AsA) and total fruit gum, decreased the content of soluble solids (SSC), and curtailed *Rhizopus* soft rot in guava fruit stored for 15 d at 8 ± 1 °C [92]. These effects may be attributed to the formation of a protective layer on the surface of the fruit through a chemical reaction between calcium chloride and lemon grass. Lemon grass has bactericidal and odor-controlling properties, and the principal component of lemon grass oil (citral) can inhibit the growth of fungal pathogens [93,94]. Furthermore, natural lemon grass extract can weaken transpiration by forming a water-retaining layer on the fruit surface, as well as repair mechanical damage, thereby reducing water loss and limiting decay during transport and storage.

### 3.3. Melatonin Treatment

Melatonin is an endogenous hormone which plays a significant role in fruit ripening and quality maintenance. Research suggests that melatonin treatment can improve postharvest quality through several mechanisms: (i) reducing respiration; (ii) increasing antioxidant capacity; and (iii) acting as a plant growth regulator to regulate ripening and senescence. Melatonin has been used to delay postharvest ripening in a diverse array of fruits, including apple [95], sweet cherry [96], banana [97,98], pear [99], peach [100], kiwifruit [85,101], and mango [102]. Exogenous melatonin treatment can also increase the disease resistance and decrease the incidence of decay in fruits during postharvest storage, as has been demonstrated in litchi [103] and strawberry [104]. Fan et al. [105] reported that exogenous melatonin treatment (600 μM, 2 h) remarkably delayed ripening, inhibited postharvest anthracnose, improved antioxidant capacity, and reduced oxidative damage in guava fruit.

### 3.4. ε-Poly-L-Lysine Treatment

ε-Poly-L-lysine (ε-PL) is a cationic homopolyamide composed of 25–30 L-lysine residues that are characterized by a unique connection between the ε-amino and α-carboxyl of L-lysine. Due to its polycationic properties, ε-PL exhibits broad-spectrum antimicrobial activity against bacteria, fungi, yeasts, and some viruses. For example, ε-PL can efficiently inhibit *Penicillium digitum* and *Botryis cinerea* [106,107]. ε-PL is also degradable, heat-stable, water-soluble, non-toxic, and environmentally friendly [108]. Therefore, ε-PL is considered a promising natural antibacterial agent, and is wildly used as a food additive in Japan, the Republic of Korea, the United States, and China [109]. Soaking guava fruit in ε-PL diluent (200 mg/L) significantly delayed quality deterioration, reduced the decay rate, and upregulates the activities of enzymatic antioxidants, and increased the contents of non-enzymatic antioxidants [110].

### 3.5. Hydrogen Peroxide Treatment

Hydrogen peroxide solution can extend the shelf-life of fruits by inhibiting the growth of microorganisms on the fruit surface at certain concentration. When hydrogen peroxide breaks down, it forms a protective O_2_ barrier on the fruit surface which prevents the growth of certain fungi. In addition, hydrogen peroxide is a strong oxidant which can both clean and disinfect fruit surface, removing impurities and harmful substances. However, high concentrations of hydrogen peroxide are harmful to fruits due to cellular oxidative damage. Juven and Pierson [111] reviewed the antimicrobial activity of hydrogen peroxide, as well as its applications in the food industry. Ismail et al. [92] reported that the use of a hydrogen peroxide solution delayed the R-type linear growth of *Rhizopus stolonifer*, a pathogen responsible for the spoilage of guava fruit. Formiga and Júnior [112] found that storing guava in an atmosphere of hydrogen peroxide had a positive effect on prolonging shelf-life and maintaining quality.

### 3.6. Acetaldehyde Treatment

Phospholipids affect the stability of cell membranes, and a reduction in phospholipids can significantly reduce membrane integrity and selective permeability. Phospholipid metabolism involves phospholipid D (PLD), lipolylacylhydrolase (LAH), lipoxygenase, and their sequential reactions. Specifically, PLD initiates membrane deterioration. Acetaldehyde is a naturally occurring volatile C6 aldehyde which strongly suppresses the activity of phospholipase D (PLD) by regulating the transcription of genes related to the phospholipase D family. In this way, acetaldehyde extends the shelf-life of fruits by delaying browning and aging.

As well as protecting membrane integrity, acetaldehyde fumigation indirectly enhances the stress resistance of fruits. It has been reported that acetaldehyde fumigation inhibits the growth of pathogenic fungi and induces the expression of genes encoding defense-related enzymes, thus minimizing the postharvest decay in banana [113]. Gill et al. [114] found that spraying guava fruit (cv. ‘Allahabad Safeda’) prior to harvest with a 0.015% *v*/*v* (1.6 mM) acetaldehyde solution was effective at preventing decay, reducing pectin methylesterase activity, and extending the shelf-life to 28 d under cold storage (6–8 °C and 90–95% RH) [114].

In summary, existing studies mainly focus on growth regulators, amino acid reagents, inorganic reagents, and their combinations (Table 4). Besides the sterilization on the surface, it mainly extends the shelf-life of guava and increases the antioxidant capacity of fruits by regulating the activity of various enzymes and the expression of their coding genes. However, due to food safety considerations, the use of chemical reagents has been greatly limited, and the screening of available reagents and efficient treatments is still in the initial stage. In addition, the pathways and functions of chemical signals in fruit are still unclear. Research on chemical treatment can be a significant reference for the selection of high-volume and low-cost fruit treatment methods, and provide new ideas and horizons for the postharvest preservation methods of other varieties of fruits.

## 4. Conclusions

**Figure 1:**
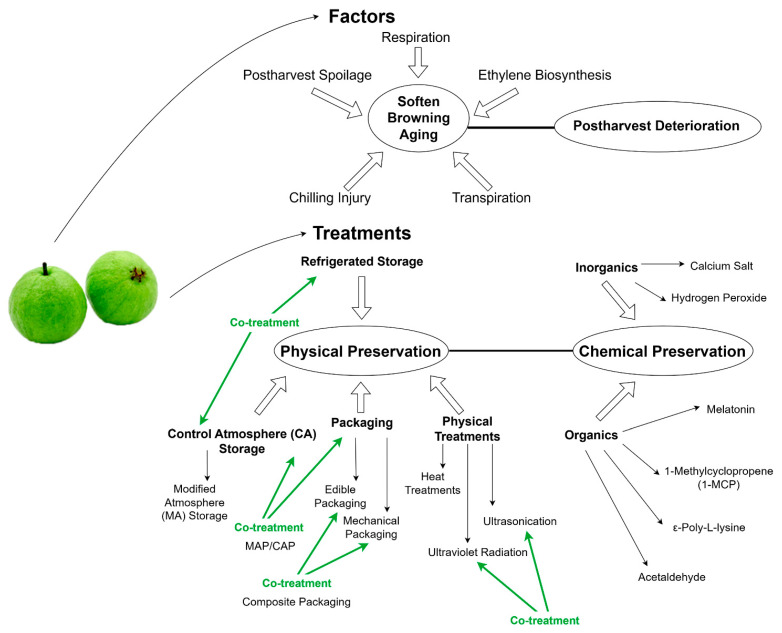
The general structure of the review, divided roughly into postharvest physiological changing factors and preservation treatments. In factors, respiration, ethylene biosynthesis, postharvest spoilage, chilling injury, and transpiration cause postharvest deterioration through softening, browning, and aging. Treatments are physical and chemical measures which can effectively prolong the shelf-life of guava. This review suggests future directions for the development of safe and effective guava preservation technologies.

Finding ways to effectively extend the shelf-life of guava fruit is imperative to support the sustainability and growth of the guava industry. Studies have revealed the physiological, biochemical, and genetic regulatory changes which occur in guava fruit during postharvest transport and storage, and have been used to develop shelf-life extension techniques (Figure 1). Researchers have developed concrete methods to extend the shelf-life of fruits, including refrigeration, special packaging, and physical and chemical treatments. However, the shelf-life of guava fruit is still limited to approximately 25 d under the most optimal storage conditions.

Emerging technologies include combination methods, edible packaging, CA storage, UV and US treatment, and chemical preservation. Expect for classical physical treatments such as packaging and CA storage, other studies are at the initial stage. However, further research is required to optimize these techniques and illuminate their precise modes of action. Available materials, ray wavelengths, and reagents, as well as their dosage, duration, and effective pathways, are prime areas for research exploration. Research ideas can be obtained from many aspects. In the former studies, reviews of guava postharvest preservation and other similar or identical families, genii, or even species are useful. Considering guava itself, studies can focus on the known quality-related enzymes and their genes, and try to summarize the signaling pathways and gene expression patterns belonging to guava by referring to other fruit varieties with more mature models. There is still a blank space to be filled in the field of postharvest preservation of guava, and research on it will be of great help in the guava industry.

## Figures and Tables

**Table 1:** MAP and CAP storage on guava.

Cultivar	Storage Condition	Main Findings	References
‘*Allahabad Safeda*’	Different packaging or wrapping materials of tissue paper, newspaper, and plastic bag at 24 °C(±2 °C) and 16 °C(±2 °C).	Lengthened the shelf-life to 12 d at room temperature. Tissue paper showed the best effect among the materials.	[38]
‘*Pant Prabhat*’	Different wrapping materials of tissue paper, cling warp, banana leaves and teak leaves, or cushioning materials of neem leaves, rice straw, and bamboo leaves at 25 ± 2 °C and 85 ± 5%RH in corrugated fibre board boxes.	Lengthened the shelf-life to at least 7 d at 25 °C, maintained the appearance and quality of fruit. Cling wrap effected the best among the materials.	[39]
‘*Lucknow-49*’	25 μm and 50 μm LDPE bags at 3% O_2_ + 5% CO_2_, 6% O_2_ + 5% CO_2_ and 9% O_2_ + 5% CO_2_, and 5 ± 1 °C and 10 ± 1 °C.	Lengthened the shelf-life to 42 d in 50 μm LDPE bags with 9% O_2_ + 5% CO_2_ at 10 °C.	[41]
‘*Hisar Safeda*’	Polythene bags (LDPE) of 200-gauge thickness by vacuum packaging at 7 ± 3 °C.	Lengthened the shelf-life to at least 21 d, maintained the physico-chemical characteristics and quality for a longer time, and delayed the ripening.	[36]
‘*Allahabad Safeda*’	LDPE, MAP with PP, MAP in LDPE with pin-hole films, MAP in PP with pin-hole films, shrink packaging with BOPP film, and vacuum packaging with PP films at ambient conditions (25 to 28 °C, 60 to 70% RH) and in a cool chamber (8 to 12 °C, 88 to 90% RH).	MAP in PP with pin-hole film in cool chamber lengthened the shelf-life to 28 d with maintenance of commercial value and biochemical quality.	[17]
‘*Pedro Sato*’	12.2 °C, low O_2_ concentration (5 kPa) and levels of CO_2_ (1, 5, 10, 15, 20 kPa)	Lengthened the shelf-life of guava to 28 d. ‘Pedro Sato’ guavas should be stored at 5 kPa O_2_ and no more than 5 kPa CO_2_, or CO_2_ damage will be caused.	[26]
‘*Allahabad Safeda*’	Polypropylene bags with 2, 4, 6, 8, and 10 pores at 6 ± 1 °C.	Lengthened the shelf-life to 25.63 d in 4-pore polypropylene bags.	[43]
Unknown	At 5 to 15 °C, 2 to 5% O_2_, and 0 to 1% CO_2_.	The best controlled condition for guava.	[27]
‘*Allahabad Safeda*’ and ‘*Lucknow-49*’	CO_2_ (20%) + O_2_ (<1%), CO_2_ (40%) + O_2_ (<1%), and CO_2_ (60%) + O_2_ (<1%) at 40 °C for 12 h then stored at 22 to 38 °C, 70 to 85% RH.	Achieved the effect of complete disinfection and lengthened the shelf-life.	[28]
‘*Paluma*’	20.9 kPa O_2_ + 0.03 kPa CO_2_ (CK),1.0 kPa O_2_ + 2.0 kPa CO_2_, 2.0 kPa O_2_ + 2.0 kPa CO_2_, 3.0 kPa O_2_ + 2.0 kPa CO_2_, 3.0 kPa O_2_ + 4.0 kPa CO_2_, and stored at 8 ± 0.2 °C, 95 ± 2.0% RH.	1.2 kPa O_2_ + 2 kPa CO_2_ effects best. Reduced the color change, firmness loss and quality loss; lengthened the shelf-life to 28 d.	[29]
Unknown (seedling guava)	Exposed to 80%:20% (N_2_O:O_2_) for 2, 4, and 6 d and stored at 20 °C.	Delayed the occurrence of decay and disease; reduced quality loss.	[30]

**Table 3:** Heat treatment on guava.

Cultivar	Treatment	Main Findings	References
‘*Gola*’ and ‘*Surahi*’	VHT at 47.5 °C for 0, 12, and 25 min	VHT at 47.5 °C for 25 min maintained the commodity quality best.	[67]
‘*Kampucea*’	HWD and VHT at 46 °C for 5, 10, 15, 20 min.	VHT 46 °C for 10 min can kill fruitfly larvae.	[64]
‘*Pedro Sato*’	HW at 47 °C for 20 min.	Effectively controlled anthracnose (*Colletotrichum simmondsii*) disease.	[65]
‘*Shweta*’	HW at 45 °C, 50 °C, 55 °C for 3 min	HW at 45 °C maintained the physiological characteristics best.	[58]
‘*Kimju*’ and ‘*Pan Srithong*’	HW at 40, 50, and 60 °C for 10 and 30 min	HW at 50 °C for 10 and 30 min successfully maintained quality and reduced microorganisms on fresh-cut guava.	[66]

**Table 4:** Chemical treatment on guava.

Cultivar	Treatment	Main Findings	References
‘*Lucknow-49*’, ‘*Allahabad Safeda*’, and ‘*Apple Colour*’	Immersed in 600 nL·L^−1^ 1-MCP aqueous solutions for 12 h.	Extended the shelf-life to 25 d at 10 °C or 9 d at 25–29 °C.	[34]
‘*Lucknow-49*’	Dipped at 2% calcium nitrate solution for 5–10 min and stored at 10 ± 10 °C with 90 ± 5% RH.	Extend the shelf-life of guava fruit to 23.83 d under cold storage; increased the TSS and sensory rating in 10 days.	[91]
Unknown	Dipped at CaCl_2_ at 2% for four minutes and fumigation with crude lemon grass oil (6 mL/carton box).	Maintained the most appearance, physical, chemical fruit properties at the sensory evaluation and freed from rots at 8 ± 1 °C for 15 d.	[92]
’*Jen-Ju Bar*’	Soaked in 0, 100, 400, and 600 μmolL^−1^ melatonin solution for 10 min and stored at 25 ± 1 °C with 70–80% RH.	Maintained the quality of guava fruit and enhanced its resistance to oxidation and disease by improving the antioxidant and defense systems of the fruit.	[105]
Unknown	Immersed in ε-PL diluent (200 mg/L) for 2 min.	Delayed the decline of quality and decay incidences; increased the activity of defense-related enzymes peroxidase and polyphenol oxidase.	[110]
‘*Pedro Sato*’	Stored in a hydrogen peroxide atmosphere.	The treatment showed positive effects on extending the shelf-life and maintaining the quality.	[112]
‘*Allahabad Safeda*’	Spraying guava fruit prior to harvest with a 0.015% *v*/*v* (1.6 mM) acetaldehyde solution.	Prevented decay, reduced pectin methylesterase activity, and extended the shelf-life to 28 d under cold storage.	[114]

## Data Availability

No new data were created or analyzed in this study. Data sharing is not applicable to this article.
